# Improved LDTW Algorithm Based on the Alternating Matrix and the Evolutionary Chain Tree

**DOI:** 10.3390/s22145305

**Published:** 2022-07-15

**Authors:** Zheng Zou, Ming-Xing Nie, Xing-Sheng Liu, Shi-Jian Liu

**Affiliations:** 1 College of Computer and Cyber Security, Fujian Normal University, Fuzhou 350117, China; zouzheng2018@fjnu.edu.cn; 2 School of Computer Science, University of South China, Hengyang 421001, China; 3 Fujian Provincial Key Laboratory of Big Data Mining and Applications, Fujian University of Technology, Fuzhou 350118, China; xingshengliu1997@hotmail.com; 4 Fujian Provincial Key Laboratory of Information Processing and Intelligent Control, Minjiang University, Fuzhou 350108, China

**Keywords:** dynamic time warping, time series, similarity evaluation, warping path, space-time complexity

## Abstract

Dynamic time warping under limited warping path length (LDTW) is a state-of-the-art time series similarity evaluation method. However, it suffers from high space-time complexity, which makes some large-scale series evaluations impossible. In this paper, an alternating matrix with a concise structure is proposed to replace the complex three-dimensional matrix in LDTW and reduce the high complexity. Furthermore, an evolutionary chain tree is proposed to represent the warping paths and ensure an effective retrieval of the optimal one. Experiments using the benchmark platform offered by the University of California-Riverside show that our method uses 1.33% of the space, 82.7% of the time used by LDTW on average, which proves the efficiency of the proposed method.

## 1. Introduction

As a common data type, time series is a sequence of discrete data obtained from a target with a fixed frequency in a period. A fundamental task regarding the time series is to measure the similarity between two given ones, which is critical to downstream works in terms of classification [1,2,3,4,5], clustering [6,7,8,9,10] and pattern recognition [11,12,13,14]. The dynamic time warping (DTW) [15] algorithm and its variants [16,17,18] are competent in similarity evaluation [19].

Given series *X* and *Y*, if they are of the same length *N*, then the similarity *S* could be described as Expression (1).

(1) $$S=\sum_{i=1}^{N}\left\|x_{i}-y_{i}\right\|$$

where $\left\|•\right\|$ stands for the Euclidean distance, $x_{i}$ and $y_{i}$ are the *i*th node of *X* and *Y*, respectively. However, more generally, the length of *X* and *Y* may not be the same. A key feature of DTW is that it can deal with two series of different lengths.

Let *N* and *M* be the length of *X* and *Y*, respectively; DTW finds the similarity by maintaining a two-dimensional cumulative distance matrix (CDM) *D* as shown in Expression (2). The algorithm calculates each element of *D* in row-major order (i.e., from left to right, from top to bottom), which starts from $d_{1,1}$ till $d_{N,M}$ according to Expression (3).

(2) $$D=\left[\begin{array}{ccc} d_{1,1} & \cdots & d_{1,M}\\ \vdots & \begin{array}{cccc} \ddots & & & \\ & d_{i-1,j-1} & d_{i-1,j} & \\ & d_{i,j-1} & d_{i,j} & \\ & & & \ddots \end{array} & \vdots \\ d_{N,1} & \cdots & d_{N,M} \end{array}\right]$$

(3) $$d_{i,j}=dis(x_{i},y_{j})+\min(d_{i-1,j},d_{i,j-1},d_{i-1,j-1})$$

where dis(•) is the distance between two nodes. After the traversal, $d_{N,M}$ will hold the value of the similarity. The matching results (or the optimal warping path in other words) could be determined according to the CDM.

**Figure 1:**
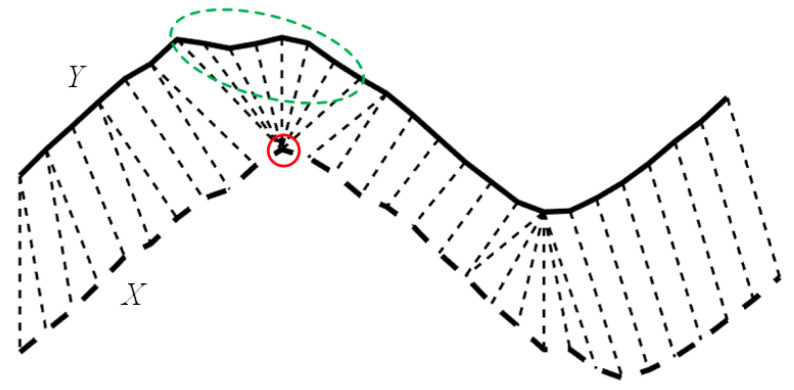
Demonstration of the pathological alignment problem of DTW, where one node in *X* (marked with the red solid circle) is matched with too many nodes in *Y* (marked within the green dotted circle).

For the evaluation of series with different lengths, as depicted in Figure 1, DTW aims to find the optimal alignment between *X* and *Y* [20], and a node in *X* may be matched with multiple nodes in *Y* (and vice versa). However, if too many nodes (marked within a green dotted circle in Figure 1) are matched with the same one (marked within a red solid circle in Figure 1) which is unreasonable in a real case, it is referred to as the well-known pathological alignment problem of DTW.

**Figure 2:**
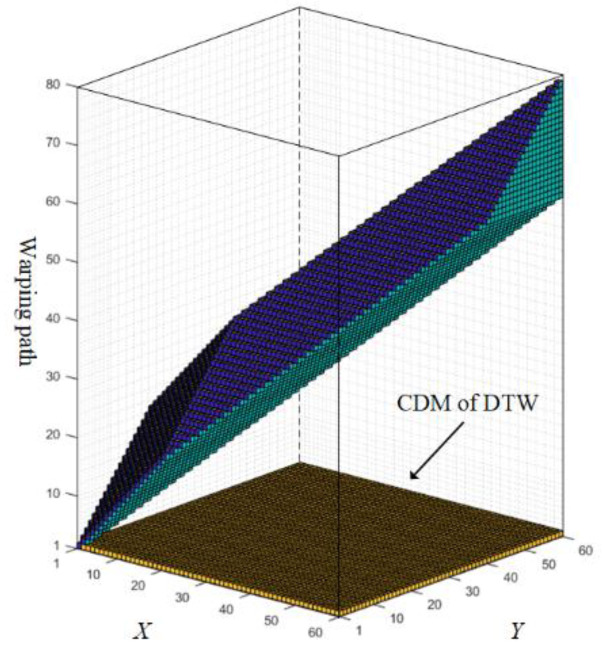
Comparison of the space and calculated amount between LDTW and DTW (tested on UCR data named SyntheticControl). The biggest cube is the CDM of LDTW, while the bottom part is the CDM of DTW.

To solve that, Zhang et al. [21] presented a state-of-the-art method named dynamic time warping under limited warping path length (LDTW). By limiting the length of the warping path in a third dimension (see Figure 2), the pathological alignment problem could be relieved. As a result, LDTW boosts the accuracy against other variants [22,23,24,25] on the benchmark platform offered by the University of California-Riverside (UCR) [26]. However, it also leads to a much higher space-time consumption.

To reduce the complexity of LDTW, an alternating matrix whose size is much smaller than the three-dimensional CDM used in LDTW is presented, and an evolutionary tree is introduced to represent the warping paths as well. The main contributions of this paper are twofold:

(1) A two-channel matrix with an alternating scheme is proposed for similarity calculation.
(2) A chain tree with an evolutionary scheme is proposed to find the optimal warping path with the similarity calculation process simultaneously.

The rest of this paper is organized as follows. The preliminary is given in Section 2. Section 3 presents the proposed method. The experiment and results are shown in Section 4. Section 5 concludes the work.

## 2. Preliminary

### 2.1. DTW

DTW is a dynamic programming algorithm for calculating the similarity of two sequences, especially those of different lengths [27]. Given time series *X* and *Y* defined by Expression (4):

(4) $$\left\{\begin{array}{c} X=\{x_{i}|1\leq i\leq N\}\\ Y=\{y_{j}|1\leq j\leq M\} \end{array}\right.$$

where *N* and *M* are the lengths of *X* and *Y,* respectively. If *P*(*X*,*Y*) defined by Expression (5) is a warping path of *X* and *Y*, each path node $p_{t}$ could be defined by a pair of nodes of *X* and *Y* as shown in Expression (6).

(5) $$P(X,Y)=\{p_{t}|1\leq t\leq L\}$$

(6) $$p_{t}=(x_{i},y_{j})(i\in [1,N],j\in [1,M])$$

In addition, the warping path also abides by the following restrains.

(1) $p_{1}=(x_{1},y_{1})$,$p_{L}=(x_{N},y_{M})$;
(2) if $p_{t}=(x_{i},y_{j})$ and $p_{t+1}=(x_{i^{\prime }},y_{j^{\prime }})$, then $0\leq i-i^{\prime }\leq 1$, $0\leq j-j^{\prime }\leq 1$.

Let $A^{X,Y}$ denote all the warping paths of *X* and *Y*, DTW aims to find an optimal one $P_{O}(X,Y)$ that possesses minimum cumulative distance as shown in Expression (7).

(7) $$\underset{P(X,Y)\in A^{X,Y}}{\min}\sum_{\left(x_{i},y_{j})\in P(X,Y\right)}dis(x_{i},y_{j})$$

where $dis(x_{i},y_{j})$ is the distance between two nodes $x_{i}$ and $y_{j}$ among a warping path *P*(*X*, *Y*).

The problem could be solved in a dynamic programming way. Namely, let $X_{e}^{s}$(or $Y_{e}^{s}$) denote the subset of *X* (or *Y*) that starts from the *s*th node to the *e*th node, the cumulative distance of $P_{O}(X_{i}^{1},Y_{j}^{1})$ consists of the node distance $dis(x_{i},y_{j})$ and the minimum value among $CD[P_{O}(X_{i-1}^{1},Y_{j}^{1})]$, $CD[P_{O}(X_{i}^{1},Y_{j-1}^{1})]$ and $CD[P_{O}(X_{i-1}^{1},Y_{j-1}^{1})]$ as described in Expression (8).

(8) $$dis(x_{i},y_{j})+\min\{CD[P_{O}(X_{i-1}^{1},Y_{j}^{1})],CD[P_{O}(X_{i}^{1},Y_{j-1}^{1})],CD[P_{O}(X_{i-1}^{1},Y_{j-1}^{1})]\}$$

where CD[•] indicates the cumulative distance of a path. This is the reason for DTW to maintain the CDM and calculate according to Expression (3), which is another version of Expression (8).

### 2.2. LDTW

To ease the pathological alignment problem, besides the series length, LDTW takes the warping path length into consideration as well, which extends the original two-dimensional CDM of size N×M to a three-dimensional matrix of size $N\times M\times L_{UB}$, where *N* and *M* are the lengths of two series, $L_{UB}$ is the upper bound of the warping path length, the range of which is [max(N,M)+1,N+M−2] under the rule of DTW (see Ref. [21] for the details about $L_{UB}$). For example, Figure 2 showed a case that applies LDTW on UCR data named SyntheticControl, where *N = M* = 60, $L_{UB}$= 79. The space used by LDTW is a cubic matrix of size 60×60×79. By contrast, DTW only uses the bottom of the cube. The elements that participated in the calculation are colored in the figure as well, which is 18490 in total for LDTW and 3600 for DTW. It shows that, compared to DTW, the time and space complexity of LDTW is greatly increased.

In this paper, a matrix of size $2\times M\times L_{UB}$ is used to replace the above three-dimensional CDM with an alternating scheme, which reduces the cost of time and space dramatically.

## 3. The Proposed Method

There are two goals for DTW and the variant algorithms in general, which are finding (1) the similarity and (2) the optimal warping path of two given time series. This section will present our solutions, respectively.

### 3.1. The Alternating Matrix Based Similarity Calculation

The primary innovation of the proposed method is the usage of a two-channel matrix with an alternating scheme, which can replace the three-dimensional CDM of LDTW and save a lot of computer memory.

**Figure 3:**
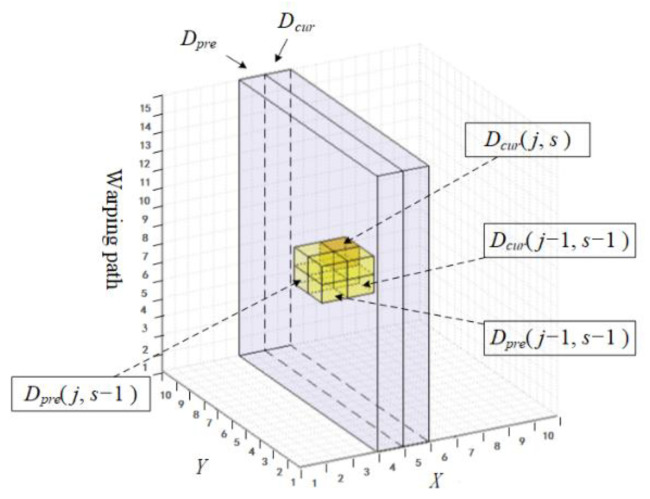
The proposed two-channel alternating matrix within the CDM space.

As illustrated in Figure 3, the proposed matrix has two channels indicated by $D_{pre}$ and $D_{cur}$, respectively. It could be seen as a subset of the three-dimensional CDM and travels over the CDM space during the similarity calculation process step by step. In each step, data in $D_{pre}$ stand for the calculated result of the previous step. Moreover, it is reserved to participate in the calculation of the current step, which happens in $D_{cur}$. The last thing to accomplish in each step is to alternate the role of the two channels, in other words $D_{cur}$(or $D_{pre}$) in Step i will be $D_{pre}$(or $D_{cur}$) in Step i+1, which is the main reason why we call our matrix the alternating matrix (AM).

**Figure 4:**
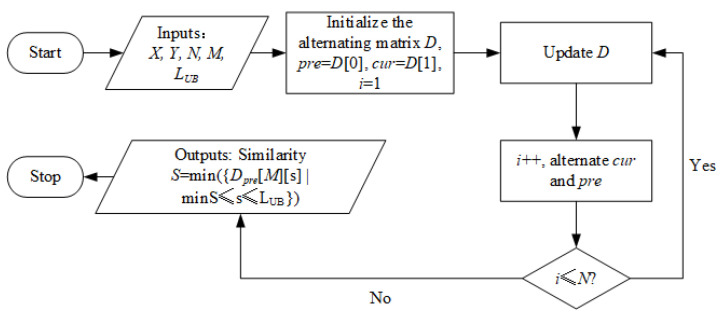
The workflow of the proposed similarity calculation process.

The calculation workflow can be seen in Figure 4. The system takes the above-mentioned $X,N,Y,M,L_{UB}$ as input and outputs the similarity *S* which equals to a specific element of the AM (i.e., $\min(\{D_{pre}[M][s]|\min S\leq s\leq L_{UB}\})$). The core step is the update of the AM, which is described in Algorithm 1. In the beginning, the algorithm travels over Y and the warping path dimension as shown from Step 1 to Step 4, where minS and maxS are the ranges calculated by functions named MinStep() and MaxStep(), respectively. Readers can find the calculation details in Ref. [21]. Step 5 specifies how an element $D_{cur}(j,s)$, as shown in Figure 3, is determined by pre-calculated $D_{pre}(j,s-1)$,$D_{pre}(j-1,s-1)$ and $D_{cur}(j-1,s-1)$. Channel $D_{pre}$ will be reset in Step 9 before the alternating process, for it will become $D_{cur}$ in the next round of iteration. The iteration stops when *i* becomes larger than *N*.
**Algorithm 1**: AM UpdateInput: *X, Y, N, M, D, i, cur, pre, L_UB_*Ouput: updated *D*1**for** *j* from 1 to *M* **do**2    *minS*←MinStep(*i*, *j*), *maxS*←MaxStep(*i*, *j*, *N*, *M*, *L_UB_*)3    **if** *minS* < *maxS* **do**4        for *s* from *minS* to *maxS* do5            
$D_{cur}[j][s]\leftarrow \min\{D_{pre}[j][s-1],D_{cur}[j-1][s-1],D_{pre}[j-1][s-1]\}$
$+dis(x_{i},y_{j})$
6        
**end for**
7    
**end if**
8**end for**9$\mathrm{reset}\;D_{pre}$

### 3.2. The Evolutionary Chain Tree Based Optimal Warping Path Determination

Besides the similarity, we can also find the corresponding warping path, which shows the matching pairs of two series. To achieve that, a chain tree with an evolutionary scheme is proposed. We also modified the structure of the AM, where each element possesses not only a value but also a pointer.

**Figure 5:**
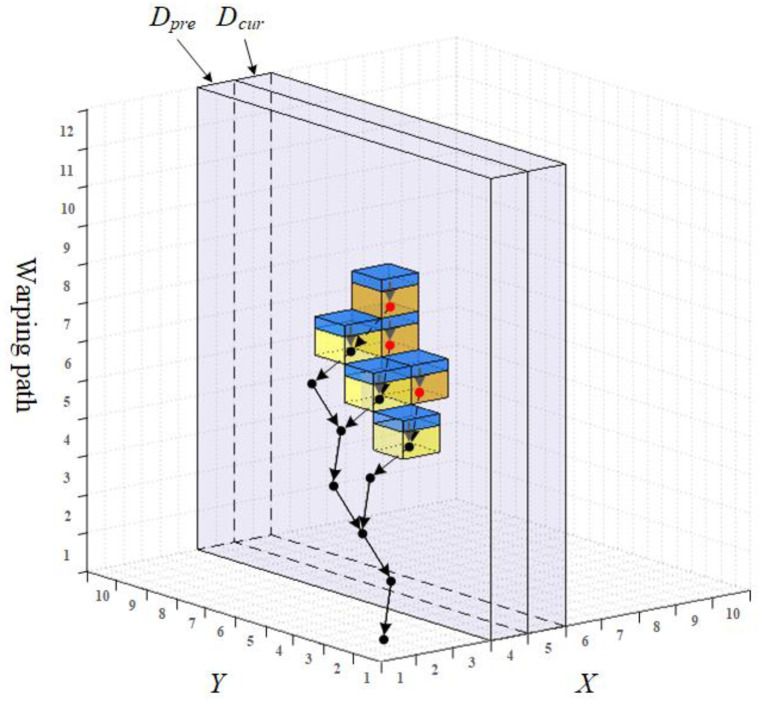
Illustration of the AM and ECT.

For example, the nodes and links of the chain tree are shown as dots and arrows in Figure 5, and six AM elements are drawn as cubes. Each cube is divided into two parts, the top part is the pointer domain leading to a corresponding tree node, while the bottom part is the value domain for the storage of the cumulative distance.

The above tree is referred to as the evolutionary chain tree (ECT) because we use a chain tree to represent the warping paths and the tree is growing and pruning dynamically during the process. The usage of ECT is another major contribution of this work.

**Figure 6:**
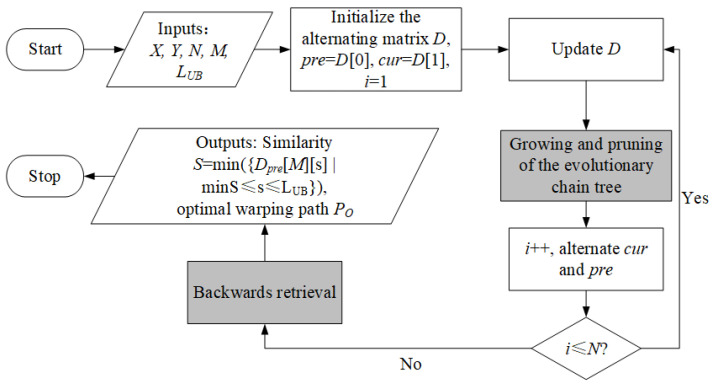
The updated workflow of the proposed method.

With the ECT, the workflow demonstrated in Figure 4 can be extended to an updated version shown in Figure 6. The main differences are marked as blocks in grey, which include the growing and pruning of the ECT, and the retrieval of the optimal warping path.

#### 3.2.1. Growing

The scale of ECT grows after each update step of AM. Specifically, as soon as the computation in $D_{cur}$ finished, tree nodes will be created and linked to the ECT. Each tree node is initialized as a structure p shown in Expression (9).

(9) p:(prior=null,data=0b0000)

where prior is the pointer that leads to a prior tree node. Description of data will be given later.

**Figure 7:**
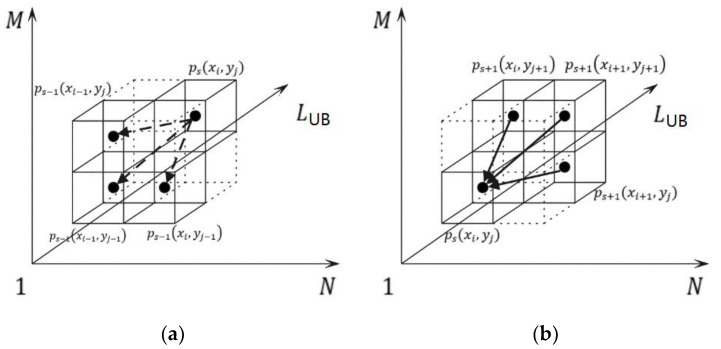
The potential precursor (**a**) and successors (**b**) of a tree node *p_s_*(*x_i_, y_j_*).

If a node $p_{s}(cur,j)$ is initialized and linked from AM element $D_{cur}(j,s)$ as shown in Figure 7a, the next question is which node is its precursor. According to Step 5 in Algorithm 1, $D_{cur}(j,s)$ is partially determined by the minimum among $D_{pre}(j,s-1)$, $D_{pre}(j-1,s-1)$ and $D_{cur}(j-1,s-1)$. Therefore, the precursor of $p_{s}(cur,j)$ is the tree node that links from the minimum among $D_{pre}(j,s-1)$, $D_{pre}(j-1,s-1)$ and $D_{cur}(j-1,s-1)$ as well. The above processes are shown in Algorithm 2, from Steps 5 to Step 7.

The data term of a tree node *p* is a four-digit value. The higher two digits are defined in Table 1, which is a clue to finding all the X and Y indexes of the optimal warping path nodes since we did not save them. Specifically, when retrieving the optimal warping path, it begins from the tree node linked from $\min(\{D_{pre}[M][s]|\min S\leq s\leq L_{UB}\})$ backwards to the first one following the pointers. Because the indexes of the last node are known, with the higher two digits, it is easy to find the indexes of the rests. While the lower two digits stand for the number of its successors, which is no more than three as shown in Figure 7b. The lower two digits are crucial to the pruning process introduced in the next section. Step 8 in Algorithm 2 describes the process related to the data term accordingly.
**Algorithm 2:** ECT GrowingInput: *N*, *M*, *D*, *i*, *cur*, *pre*, *L_UB_*Ouput: updated *D*1**for** *j* from 1 to *M* **do**2    *minS*←MinStep(*i*, *j*), *maxS*←MaxStep(*i*, *j, N, M, L_UB_*)3    **if** *minS* < *maxS* **do**4        **for** *s* from *minS* to *maxS*
**do**5
             initialize p:(prior=null,data=0b0000)
$,\;D_{cur}[j][s].ptr\leftarrow p$
6            *q*←min{*D_pre_ [j][s*−1*], D_pre_ [j-*1*][s*−1*], D_cur_ [j-*1*][s*−1*]*}7            p.prior←q.ptr
8            
$p.data\leftarrow \left\{\begin{array}{l} \begin{array}{cc} 0b0100, & ifq=D_{pre}[j][s-1] \end{array}\\ \begin{array}{ccc} 0b1000, & ifq=D_{cur}[j-1][s-1] & ,\;p.prior.data++ \end{array}\\ \begin{array}{cc} 0b1100, & ifq=D_{pre}[j-1][s-1] \end{array} \end{array}\right.$
9        
**end for**
10    
**end if**
11**end for**

#### 3.2.2. Pruning

**Figure 8:**
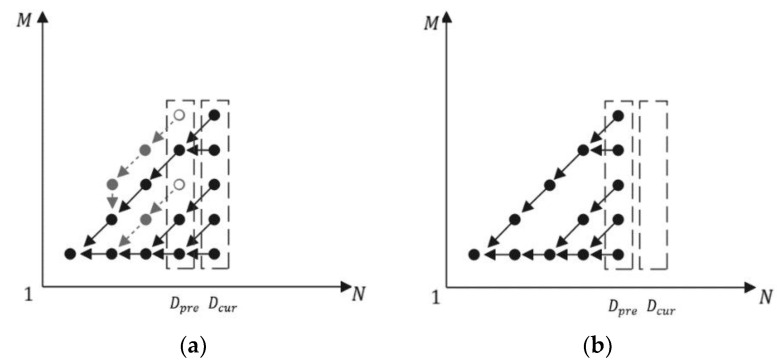
Illustration of ECT before (**a**) and after (**b**) pruning.

As the ECT grows, some branches lose their activity. Figure 8a demonstrates such a case, where two branches are not growing after new nodes have been added to ECT. Those branches can be pruned to save memory; the pruning result is shown in Figure 8b.

In our method, the pruning starts from leaf nodes drawn as circles in Figure 8a. They can be found from $D_{pre}$ as shown in Algorithm 3, Step 5. If their lower two-digit data term equals 0b00, then they need to be removed because it means they have no successor.
**Algorithm 3:** ECT PruningInput: *N, M, D, i, cur, pre, L_UB_*Ouput: updated *D*1**for** *j* from 1 to *M* **do**2    *minS*←MinStep(*i*−1, *j*), *maxS*←MaxStep(*i*−1, *j, N, M, L_UB_*)3    **if** *minS* < *maxS* **do**4        **for** *s* from *minS* to *maxS*
**do**5            $p\leftarrow D_{pre}[j][s].ptr$
6            **while** lower(*p*.*data*) equal to 0b00 **do**7                *q*←*p*, *p*←*p*.*prior*, *p.data*--, delete *q*8            
**end while**
9        
**end for**
10    
**end if**
11**end for**

**Figure 9:**
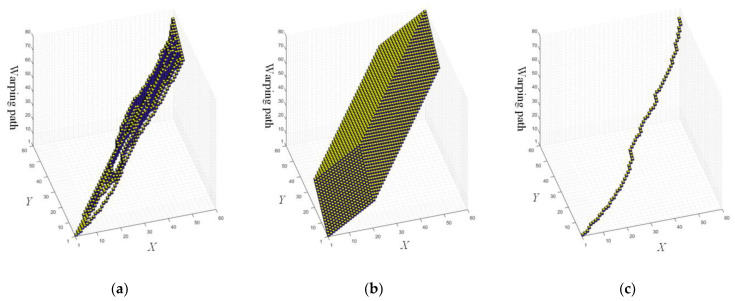
The final ECT (**a**) with pruning and (**b**) without pruning. (**c**) The optimal warping path that extracted from the final ECT.

Figure 9a shows the final ECT applying the proposed method on SyntheticControl. Moreover, if no pruning is used, it would look like the one shown in Figure 9b. Figure 9c shows the optimal warping path.

## 4. Experiments and Results

The proposed method was implemented using the C++ programming language. The public dataset UCR [26] was adopted for the 1-NN classification tests on a desktop computer with AMD Ryzen 7 5800X 3.80 GHz CPU, 64 GB memory. We compared our method with LDTW in terms of time and space consumption.

### 4.1. Comparisons

To compare our method with LDTW in space costs, we tested it on all species in UCR. We selected the result of 15 data points for showing, and each is of a different name and length as described in the first and second columns of Table 2. There are two key phases in our method, namely the similarity calculation phase and optimal warping path determination phase, therefore we recorded the space cost of them as Ph1(MB) and Ph2(MB). As the table shows, our method uses 1.33% of the space used by LDTW on average.

**Figure 10:**
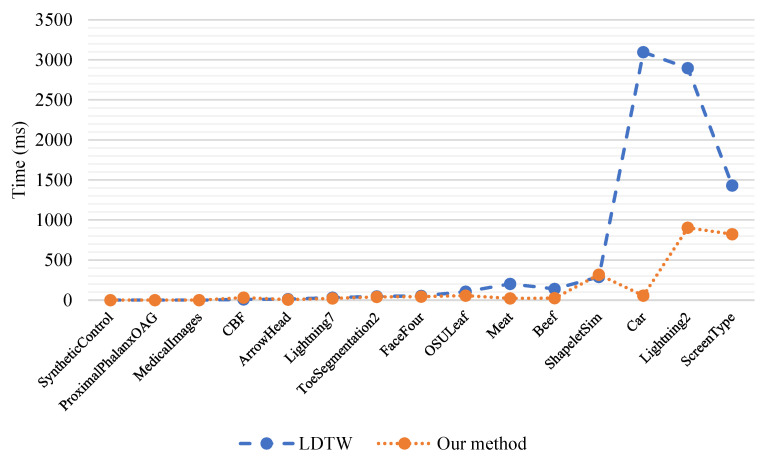
Comparisons between LDTW and our method in terms of time costs.

Our comparison was also completed in time costs. According to the results shown in Figure 10, there are 15 data points which are organized in ascending order of scale in the first column of Table 2 in the horizontal direction, and there are the specific time costs (ms) of our method and LDTW in the vertical direction. As the scale of the time series increases from left to right, the superiority of our method becomes more obvious.

### 4.2. Ablation Experiment

**Figure 11:**
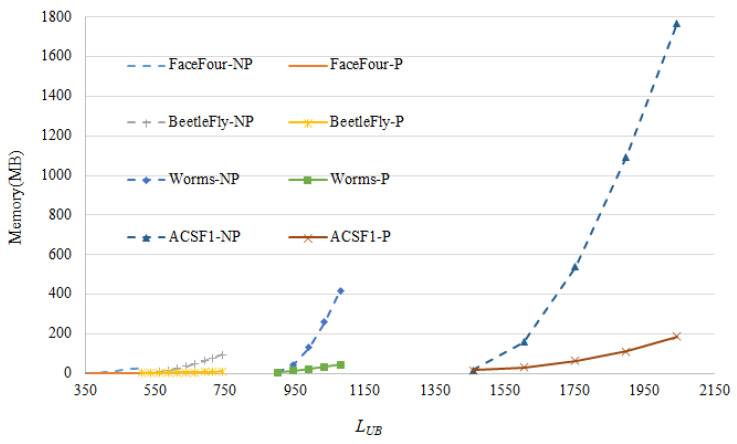
The ablation experiment results. The horizontal axis is the parameter $L_{UB}$, the vertical axis is the memory cost running on different data. *-P and *-NP stand for the method with and without pruning, respectively.

To show the contribution of the pruning proposed in our method, system performance with and without pruning is investigated. As Figure 11 shows, the space consumption could be greatly reduced with the pruning process. In addition, it is normal that the space cost rises along with the increase of parameter $L_{UB}$. With the help of pruning, few variations have been found in Figure 11, compared to the case without pruning which is sensitive to the choice of $L_{UB}$. The scales of the data used in Figure 11 are listed in Table 3.

## 5. Discussion

Thanks to the proposed alternating matrix, great achievement has been made in reducing the memory cost compared to the LDTW method. The price of this huge deflation is the need for an additional data structure to maintain the warping paths, as well as a new strategy for optimal warping path retrieval. We solve that problem by the proposed evolutionary chain tree, which will sacrifice little time and space, but it is just a drop in the ocean compared to the contributions. The performance of the proposed method still outranges the LDTW a lot.

Another issue is about the choice of $L_{UB}$, which is the only parameter in this method. The usage and setting criteria of $L_{UB}$ in our work follow the idea introduced by the LDTW algorithm [21]. In experiments, we found that different values of $L_{UB}$ may slightly alter the accuracy, but it is insensitive to our final space costs as shown in the ablation experiment. Therefore, to get a fairer comparison, we adopted the same method as [21] for $L_{UB}$ to keep a similar parameters environment.

## 6. Conclusions

This paper proposes a novel resolution for recording and exploding wrapping paths with much less space-time complexity. Firstly, a two-channel matrix is created and travels over the entire cumulative distance space with an alternating scheme to calculate the similarity. Secondly, a chain tree is involved to record all warping paths, and the tree is gradually growing and pruned along with the matrix alternating simultaneously, which ensures an efficient retrieval of the optimal path. Experiments running on the UCR benchmark show that our method uses 1.33% of the space, 82.7% of the time used by LDTW on average. Future work would focus on improving the evaluation accuracy.

## Figures and Tables

**Table 1:** Definition of the higher two-digit data term for tree node *p_s_*(*cur, j*).

	$p_{s-1}(pre,j)$	$p_{s-1}(cur,j)$	$p_{s-1}(pre,j-1)$
$p_{s}(cur,j)$	0b01	0b10	0b11

**Table 2:** Comparisons between LDTW and our method in terms of space costs.

Data Name	Length	LDTW(MB)	Our Method		
			Ph1 (MB)	Ph2 (MB)	Total (MB)
SyntheticControl	60	1.02	0.03	0.01	0.04
ProximalPhalanxOAG	80	1.95	0.05	0.00	0.05
MedicalImages	99	3.89	0.08	0.01	0.09
CBF	128	12.75	0.20	0.25	0.45
ArrowHead	251	60.32	0.48	0.00	0.48
Lightning7	319	132.76	0.83	0.13	0.96
ToeSegmentation2	343	169.64	0.99	0.31	1.30
FaceFour	350	180.85	1.03	0.25	1.29
OSULeaf	427	321.33	1.51	0.47	1.98
Meat	448	343.00	1.53	0.00	1.53
Beef	470	400.27	1.70	0.02	1.72
ShapeletSim	500	567.44	2.27	0.91	3.18
Car	577	755.66	2.62	0.12	2.74
Lightning2	637	1211.99	3.81	4.40	8.21
ScreenType	720	1657.18	4.60	15.39	19.99

**Table 3:** The scales of the data shown in Figure 11.

**Data**	FaceFour	BeetleFly	Worms	ACSF1
**Scale**	350 × 350	512 × 512	900 × 900	1460 × 1460

## Data Availability

The data presented in this study are available on request from the corresponding author.
